# The 20-year outcome of ascending aorta replacement and total arch replacement comparison in DeBakey type I acute aortic dissection

**DOI:** 10.1097/JS9.0000000000003530

**Published:** 2025-10-18

**Authors:** Chun-Yang Huang, Chiao-Po Hsu, Ying-Ting Kuo

**Affiliations:** a Department of Medicine, School of Medicine, National Yang-Ming Chiao-Tung University, Taipei, Taiwan; b Division of Cardiovascular Surgery, Department of Surgery, Taipei Veterans General Hospital, Taipei, Taiwan; c Division of Cardiovascular Surgery, Department of Surgery, National Yang-Ming Chiao-Tung University Hospital, Yilan, Taiwan; d Department of Radiology, Taipei Veterans General Hospital, Taipei, Taiwan

**Keywords:** acute aortic dissection, age ≧ 65 years, ascending aorta replacement, DeBakey type I, total arch replacement

## Abstract

**Background:**

The mortality rate of acute DeBakey type I aortic dissection increases by 1%–2% every hour after the first presentation if left untreated. However, the long-term outcomes of ascending aorta replacement (AR) versus total arch replacement (TAR) remain unclear. This study evaluated and compared the long-term outcomes between AR and TAR.

**Materials and Methods:**

This retrospective study included 398 patients with acute DeBakey type I aortic dissection who underwent surgical repair between March 2002 and October 2024. Patient data were extracted from medical records, and patients were stratified into two groups (AR and TAR groups) according to the surgical procedure. Inverse probability of treatment weighting was applied for further analysis.

**Results:**

Higher incidences of acute kidney injury (32.3% vs 20.6%, *P* = 0.029), new-onset stroke (9.5% vs 3.7%, *P* = 0.016), and bleeding (30.1% vs 16.2%, *P* = 0.007) and longer hospital stays (34.4 vs 25.7 days, *P* = 0.042) were observed in the TAR group than in the AR group immediately postoperation. In long-term follow-up, no significant differences were observed between the groups regarding reintervention or mortality events. In addition, the risk of relative mortality associated with TAR was more apparent in patients older than 65 years.

**Conclusion:**

No significant differences were observed between TAR and AR in terms of 30-day mortality, reintervention events, aorta-related mortality, or overall mortality under the tear-oriented policy for acute DeBakey type I aortic dissection. Notably, older patients undergoing TAR had poorer overall survival outcomes than did those undergoing AR, particularly among patients older than 65 years.

**HIGHLIGHTS**

- Higher postoperative complications but no significant difference in 30-day mortality in TAR compared with AR.
- No significant differences in reintervention events, aorta-related mortality, or overall mortality between TAR and AR under 65 years.
- TAR has a poorer overall survival outcome than those undergoing AR among patients older than 65 years.

## Introduction

Acute DeBakey type I aortic dissection is a critical cardiovascular condition with a mortality rate that increases by 1%–2% per hour after initial presentation if left untreated[1]. Prompt surgical repair is therefore required after a diagnosis of acute aortic dissection is made[2]. Surgical management is associated with considerably lower in-hospital mortality than is conservative treatment,^[3-6]^ and long-term survival and quality of life after surgery are generally acceptable. Tear-oriented surgery has been widely recommended for the management of acute DeBakey type I aortic dissection. Two surgical methods were applied, that is, limited and extensive aortic repair. A focused PubMed literature review and meta-analysis were conducted to compare these two methods. Several knowledge gaps remain. First, some studies have reported that extensive aortic surgery leads to better long-term outcomes without increasing mortality and has a low incidence of late aortic complications.^[7–9]^ By contrast, other studies have indicated that extensive aortic surgery may result in severe complications, such as bleeding, low cardiac output syndrome, stroke, prolonged ventilator dependence, and dialysis.^[10–12]^ The long-term benefits of the surgery remain unclear^[13,14]^. Second, older age is a strong independent risk factor for hospital mortality in patients undergoing cardiovascular surgery[15], but studies on older patients have not provided sufficient data to enable a comparison of the long-term outcomes between limited and extensive aortic surgeries during follow-up. Third, inconsistent patient selection across studies limits the comparability of their reported outcomes. Achieving a balance between immediate lifesaving intervention and optimal long-term results remains, and several key concerns require clarification.

To address these gaps, the present study applied inverse probability of treatment weighting (IPTW) to minimize confounding. It evaluated the preoperative, perioperative, immediate postoperative, and long-term outcomes of limited and extensive aortic repair in patients with acute DeBakey type I aortic dissection. In particular, we examined the long-term outcomes of and the effect of age in the two surgical approaches.

## Methods

This retrospective observational study included 398 patients with acute DeBakey type I aortic dissection who underwent surgical repair between March 2002 and October 2024. The patients were divided into two groups: those who underwent ascending aorta replacement (AR) accompanied by total arch replacement (TAR) and those who received only ascending AR without TAR. AR was performed when the primary tear was located in the proximal aorta, whereas TAR was recommended when the primary tear was located in the arch. For a primary tear in the descending aorta, thoracic stent grafts were deployed from the landing zone of the arch graft, or the arch-sparing method was used, as described previously.^[15,16,17]^ AR was defined as replacement of the ascending aorta, including repair of the root or lesser curvature of the arch. TAR was defined as reconstruction of the entire arch through direct vascular grafting or hybrid stent grafting. Reconstruction of the supra-aortic branches included debranching to the ascending aorta graft or *in situ* reconstruction. Malperfusion of visceral branches was treated through endovascular branch stenting. Immediate postoperative complications were recorded. Acute renal injury was defined as renal function deterioration requiring continuous venovenous hemofiltration support after the exclusion of preoperative kidney malperfusion. New-onset stroke was defined as the presence of both clinical symptoms and radiologic evidence within 30 days postoperatively, and patients with prior cerebrovascular accident or preoperative cerebral malperfusion were excluded. Clinically significant symptoms were considered to be those persisting for over 24 hours and confirmed by a neurologist. Radiologic evidence was routinely assessed using CT imaging. MRI was not used routinely because of concerns related to unstable hemodynamics and bleeding risk during the scan[18]. Bowel ischemia was confirmed through CT imaging and consultation with a general surgeon after the exclusion of preoperative small bowel malperfusion. Postoperative bleeding was defined as bleeding requiring reoperation for hemostasis within 48 hours after the primary surgery. The demographic characteristics, clinical and perioperative variables, postoperative complications, and follow-up findings were compared between the two groups.

### Ethical considerations

Because all patient data retrieved from medical and surgical records were deidentified after April 2022 and provided to the research group anonymously from September 2022, the IRB waived the requirement for signed informed consent. Data supporting the findings of this study are available upon request from the corresponding author. These data are not publicly available because of privacy and ethical restrictions.

### STROCSS guideline

The work has been reported in line with the STROCSS criteria[19].

### Surgical procedures

Tear location was determined using preoperative computed tomography angiography (CTA). A tear was classified as being in the ascending aorta if confined to the tubular ascending aorta and as arch-related if located between the innominate artery and left subclavian artery. Multiple tears were recorded accordingly. Tears in the ascending aorta or lesser curvature of the arch were completely resected during ascending AR, whereas those in the greater curvature of the arch were resected during TAR. If tear location could not be determined by CTA, intraoperative inspection was used to finalize the location and guide surgical decision-making.

The right axillary and common femoral arteries were exposed and looped. Vascular grafts measuring 8 mm were anastomosed to the arteries in an end-to-side manner. Double-arterial cannulas were connected to the 8-mm vascular grafts. A single venous cannula was placed over the right atrium after full sternotomy. Subsequently, the ascending aorta was clamped, and cardiac arrest was induced using antegrade cardioplegia infusion. The ascending aorta was then opened, and the pathological tissue was resected. After the rectal temperature was cooled to the target level, systemic circulation was arrested, and the supra-aortic vessels were clamped. The axillary arterial cannula supplied continuous cold blood perfusion (15°C, 10–20 mL/kg/min) to the brain tissue through antegrade cerebral perfusion (ACP) under a perfusion pressure of 50 mmHg. Once brain protection was ensured, the aortic clamp was released, and the arch intima was inspected for a primary tear. Arch replacement was indicated when the primary tear was located at the great curvature, and a four-branched vascular graft was applied for TAR. In Taiwan, thoracic stent grafting became broadly applied after 1 June 2011, when public health insurance began covering the procedure. Hybrid TAR was subsequently performed more frequently if malperfusion or a visceral vessel originating from the false lumen was suspected. Before stent grafting for the descending aorta commenced, an elephant trunk vascular graft was placed inside the arch. The proximal edge of the elephant trunk graft was sutured to the proximal arch edge with Teflon felt by using the sandwich method. The thoracic stent graft was deployed at the distal edge of the elephant trunk graft in an antegrade manner, with 10% oversizing under direct vision. Next, the distal part of the four-branched vascular graft was sutured to the proximal edge of the elephant trunk graft. After the completion of the distal anastomosis, systemic perfusion was resumed, and the patient was rewarmed. The proximal and branch anastomoses of the four-branched graft were performed to reconstruct the ascending aorta and supra-aortic vessels. The proximal part of the four-branched graft was anastomosed to the proximal segment of the ascending aorta in an end-to-end manner. The first and second branches of the graft were individually anastomosed to the innominate and left carotid arteries in an end-to-side manner. The third branch of the graft was anastomosed to the left subclavian artery in an end-to-side manner, and the area around the left subclavian artery was exposed. When the arch intima was intact, a straight vascular graft was applied for AR. Thoracic stent grafting was considered before distal anastomosis was employed if preoperative malperfusion was suspected. The thoracic stent was deployed distal to the orifice of the subclavian artery in an antegrade manner, with 10% oversizing under direct vision. Subsequently, the proximal and distal anastomoses of the aorta were repaired using Teflon felt through the sandwich method. After the completion of the reconstruction, the aortic clamp was removed, and cardiac activity was restored.

### Surveillance protocol

Major postoperative complications and hospital mortality were recorded. All surviving patients were followed regularly and underwent CT surveillance. CT imaging was performed using a HiSpeed Advantage scanner (GE Healthcare, Waukesha, WI, USA) with a collimation of 5 mm, a table speed of 10 mm/s, a pitch of 2.0, and a tube current of 120 kV and 230–250 mA. The first CT scan was conducted within 1 month after surgery, followed by a second scan at 6 months, and annual scans were performed thereafter. Patients were required to visit the clinic every 3 months after discharge, and all CT images were archived for further evaluation. Reintervention was defined as any surgery performed because of residual aortic disease or complications arising from the primary surgery after the first discharge.

## Calculations

All variables presented in tables are expressed as means ± standard deviations. The chi-square test was used to compare nominal variables, and the Mann–Whitney *U* test was applied to compare continuous variables between the groups. IPTW based on the surgical method and derived from the propensity score was used to balance the distribution of the demographic and preoperative characteristics between the TAR and AR groups. The propensity score represented the probability of being allocated to the TAR group and was derived from a multivariable logistic regression model. All covariates listed in Table 1 were treated as explanatory variables. Extreme weights were truncated at the 99th percentile. The risk of overall mortality or aorta-related mortality events between the groups was compared using the Cox proportional hazards model. The incidence of distal or proximal reintervention events between groups was compared using the Fine and Gray subdistribution hazards model, which accounts for overall mortality as a competing risk. To evaluate unmeasured confounding, *E* values for the observed hazard ratios (HRs) were calculated. The interaction between surgical approach and time period was assessed using weighted Cox models. A *P*-value of <0.05 was considered significant. An IPTW-adjusted Cox proportional hazards model incorporating an interaction term for age and surgical method was used to investigate the age-dependent effect of surgery type on overall mortality risk. Continuous age was modeled as a flexible restricted cubic spline with three knots located at the 10th, 50th, and 90th percentiles. HRs (TAR relative to AR) and their 95% confidence intervals were obtained for all age groups. The analysis was conducted using R, version 4.4.3 (Lucent Technologies, Union, NJ, USA). A *P*-value of less than 0.05 was considered significant.

**Table 1:** Demographic and preoperative status of the study population stratified by surgery

	Before IPTW			After IPTW		
	AR, *N* = 243	TAR, *N* = 153	*P*-value	AR, *N* = 390.6	TAR, *N* = 346.6	*P*-value
Demographic						
Age, years, (mean ± SD)	59.3 ± 13.5	58.3 ± 12.5	0.484	58.8 ± 13.2	59.0 ± 13.3	0.885
Age ≧ 65 years	84(34.6)	46(30.1)	0.413	125.1 (32.0)	122.1 (35.2)	0.591
Female	90(37.0)	41(26.8)	0.046	134.2 (34.4)	109.5 (31.6)	0.639
Hypertension	195(80.2)	113(73.9)	0.172	312.2 (79.9)	277.9 (80.2)	0.950
Smoking	60(24.7)	49(32.0)	0.140	105.9 (27.1)	95.2 (27.5)	0.949
CAD	30(12.3)	17(11.1)	0.833	45.2 (11.6)	39.4 (11.4)	0.959
Hyperlipidemia	20(8.2)	11(7.2)	0.855	35.0 (9.0)	24.3 (7.0)	0.574
Diabetes	24(9.9)	14(9.2)	0.949	40.1 (10.3)	34.6 (10.0)	0.942
CVA	19(7.8)	14(9.2)	0.779	37.3 (9.5)	35.1 (10.1)	0.892
Chronic renal disease	8(3.3)	10(6.5)	0.207	16.2 (4.1)	16.4 (4.7)	0.813
Hemodialysis	1(0.4)	4(2.6)	0.147	1.0 (0.3)	4.0 (1.2)	0.141
Marfan syndrome	13(5.3)	5(3.3)	0.471	16.2 (4.2)	10.5 (3.0)	0.565
Preoperative malperfusion status						
Cardiogenic shock	49(20.2)	25(16.3)	0.413	80.8 (20.7)	75.9 (21.9)	0.826
Cerebral	23(9.5)	26(17.0)	0.040	57.9 (14.8)	56.2 (16.2)	0.767
Upper limbs	3(1.2)	1(0.7)	0.963	3.4 (0.9)	1.4 (0.4)	0.514
Celiac	11(4.5)	9(5.9)	0.716	16.9 (4.3)	15.2 (4.4)	0.978
SMA	7(2.9)	4(2.6)	1.000	10.3 (2.6)	9.8 (2.8)	0.908
RA	24(9.9)	11(7.2)	0.462	36.8 (9.4)	27.8 (8.0)	0.687
Lower limbs	19(7.8)	12(7.8)	1.000	27.8 (7.1)	29.8 (8.6)	0.664

CAD, coronary artery disease; CVA, cerebrovascular accident; SMA, superior mesenteric artery; RA, renal artery; IPTW, inverse probability of treatment weighting.

*P* ≦ 0.05, statistical significance.

## Results

**Figure 1.**
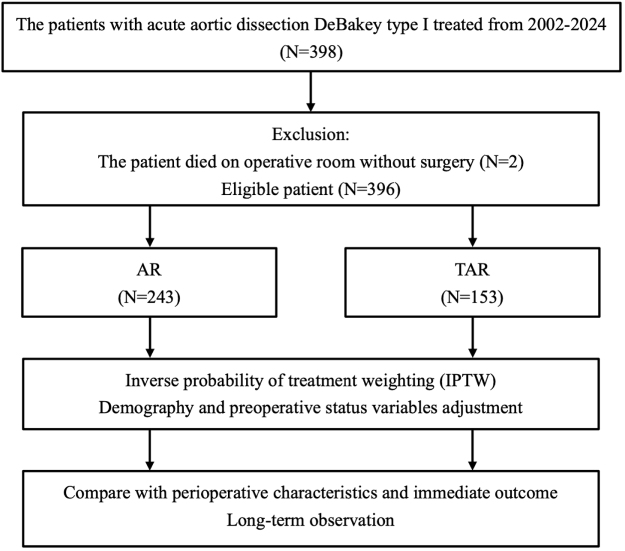
Flow chart of patients undergoing aortic dissection repair.

Surgery was performed for acute DeBakey type I aortic dissection in 396 patients, with 243 having undergone AR and 153 having undergone TAR (Fig. 1). The TAR group had a lower proportion of male patients than did the AR group (26.8% vs 37.0%, *P* = 0.046; Table 1). Regarding preoperative status, a higher incidence of cerebral malperfusion was observed in the TAR group (17.0% vs 9.5%, *P* = 0.040; Table 1). Meanwhile, cardiogenic shock was observed 20.2% in AR and 16.3% in TAR. Regarding perioperative characteristics, the TAR group had a higher primary tear resection rate (85.0% vs 62.6%, *P* = 0.001) and a higher thoracic stent grafting rate (81.0% vs 17.7%, *P* = 0.001) than did the AR group. The TAR group also had longer durations of cardiopulmonary bypass (311.3 vs 261.5 min, *P* = 0.001), aortic clamping (164.5 vs 131.7 min, *P* = 0.001), and circulatory arrest with ACP (50.3 vs 41.5 min, *P* = 0.001) compared with the AR group (Table 2). After emergency aortic repair, the TAR group had a higher incidence of acute kidney injury (AKI; 32.7% vs 21.8%, *P* = 0.022), new-onset stroke (10.5% vs 4.5%, *P* = 0.038), and bleeding (26.1% vs 16.9%, *P* = 0.036) and longer hospital stays (36.4 vs 27.0 days, *P* = 0.007) than did the AR group (Table 2). No significant differences were noted between the groups in the incidence of bowel ischemia or 30-day mortality. Among the 324 surviving patients, 202 were in the TAR group and 122 were in the AR group (Table 2). In 3- to 6-month follow-up imaging, the AR group had a 43.1% incidence of a patent false lumen at the arch level, whereas the TAR group had a 49.2% incidence at the descending aorta (Table 2).

**Table 2:** Perioperative characteristics and immediate postoperative outcome in the IPTW-adjusted cohort

	Before IPTW			After IPTW		
	AR, *N* = 243	TAR, *N* = 153	*P*-value	AR, *N* = 390.6	TAR, *N* = 346.6	*P*-value
Perioperative characteristics						
Entry site of tear (As/Ar/Retro/Un)	136/15/69/23 (56.0/6.2/28.4/9.5)	15/110/27/1 (9.8/71.9/17.6/0.7)	<0.001	233.3/24.6/106.6/26.1 (59.7/6.3/27.3/6.7)	38.9/243.7/55.2/8.8 (11.2/70.3/15.9/2.5)	<0.001
Entry site in Arch zone (1/2/3)	14/4/8	13/32/68	–	27.9/5.8/12.9	46.1/67.9/138.0	–
Primary tear size, mm	12.6 ± 4.9	13.3 ± 5.5	0.197	12.6 ± 4.8	13.2 ± 4.9	0.275
Proximal residual location (Ar/Ds/Ab/Un)	26/93/73/51 (10.7/38.3/30.0/21.0)	0/59/53/41 (0/38.6/34.6/26.8)	<0.001	46.7/156.7/115.6/71.6 (12.0/40.1/29.6/18.3)	0/140.0/114.7/91.9 (0/40.4/33.1/26.5)	<0.001
Proximal residual tear size, mm	9.3 ± 4.4	8.4 ± 4.1	0.059	9.1 ± 4.5	8.1 ± 3.7	0.056
DsAo diameter, mm	33.2 ± 5.4	33.2 ± 7.1	0.940	32.9 ± 5.2	32.8 ± 6.3	0.969
DsAo FL diameter, mm	16.7 ± 8.1	17.3 ± 8.9	0.451	16.5 ± 7.7	16.7 ± 7.9	0.779
DsAo ≧ 3.5 cm	68(28.0)	43(28.1)	10.000	101.7(26.0)	82.1(23.7)	0.643
DeBakey type extent (Ds/Ab/Ili)	24/75/144 (9.9/30.9/59.3)	16/39/98 (10.5/25.5/64.1)	0.515	37.1/108.3/245.2 (9.5/27.7/62.8)	40.1/87.6/217.9 (11.8/25.3/62.9)	0.789
Tear resection	152(62.6)	130(85.0)	<0.001	252.5(64.6)	299.1(86.3)	<0.001
Concomitant surgery						
Bentall	32(13.2)	26(17.0)	0.367	52.3 (13.4)	49.1 (14.2)	0.847
David	3(1.2)	2(1.3)	10.000	3.4 (0.9)	3.2 (0.9)	0.934
CABG	7(2.9)	4(2.6)	10.000	7.8 (2.0)	14.8 (4.3)	0.289
Aortic valve surgery	39(16.0)	11(7.2)	0.015	58.3 (14.9)	32.6 (9.4)	0.212
TAA in DsAo	43(17.7)	124 (81.0)	<0.001	93.9 (24.1)	230.0 (66.4)	<0.001
Isolated IA bypass	12 (4.9)	0	0.013	25.0 (6.4)	0	0.001
CPB, min	261.5 ± 69.8	311.3 ± 91.5	<0.001	262.0 (68.8)	316.6 (89.0)	<0.001
Clamp, min	131.7 ± 42.9	164.5 ± 50.2	<0.001	129.6 (41.2)	168.1 (55.4)	<0.001
DHCA, min	41.5 ± 19.0	50.3 ± 29.9	<0.001	40.7 (18.7)	56.3 (32.2)	<0.001
Immediate postoperative outcome						
AKI	53(21.8)	50(32.7)	0.022	80.6 (20.6)	111.9 (32.3)	0.029
New onset stroke	11(4.5)	16(10.5)	0.038	14.4 (3.7)	32.9 (9.5)	0.016
Ischemic bowel	6(2.5)	7(4.6)	0.385	9.6 (2.5)	13.8 (4.0)	0.422
Bleeding	41(16.9)	40(26.1)	0.036	63.1 (16.2)	104.4 (30.1)	0.007
30-days mortality	41(16.9)	31(20.3)	0.473	62.7 (16.1)	89.6 (25.8)	0.061
Admission, days	27.0 ± 30.2	36.4 ± 38.5	0.007	25.7 ± 27.1	34.4 ± 39.6	0.042
Postop follow-up	*N* = 202	*N* = 122		*N* = 327.9	*N* = 257.0	
Patent false lumen level						
Ar/Ds/Abd or thromb	87/35/80 (43.1/17.3/39.6)	10/60/52 (8.2/49.2/42.6)	<0.001	146.3/61.8/119.8 (44.6/18.9/36.5)	18.8/122.6/115.7 (7.3/47.7/45.0)	<0.001
Mean follow-up, year	7.6 ± 6.0	5.3 ± 4.4	<0.001	7.0 ± 5.5	6.2 ± 5.1	0.341
Distal reintervention indication						
Residual dissecting aneurysm	22 (9.1)	0	–	29.6 (7.6)	0.0 (0.0)	–
SINE	2 (0.8)	3 (2.0)		3.3 (0.8)	4.5 (1.3)	
Type I endoleak	1 (0.4)	0		3.0 (0.8)	0.0 (0.0)	
Stent deformity	1 (0.4)	1 (0.7)		1.8 (0.5)	1.2 (0.3)	
Distal anastomosis leakage	7 (2.9)	0		19.3 (4.9)	0.0 (0.0)	
Mycotic aneurysm	1 (0.4)	0		1.1 (0.3)	0.0 (0.0)	
Patent false lumen from distal reentry	1 (0.4)	6 (3.9)		1.1 (0.3)	28.5 (8.2)	
Complete previous ET to FET	0	2 (1.3)		0.0 (0.0)	9.0 (2.6)	

Ds or DsAo, descending aorta; As, ascending aorta; Ar, arch; Ab, abdominal; Ili, iliac; Retro, retrograde; Un, unknown; thromb, thrombosis; CABG, coronary artery bypass graft; TAA, thoracic stent graft; AKI, acute kidney injury; CPB, cardiopulmonary bypass; DHCA, deep hypothermia circulatory arrest; SINE, stent graft-related new entry; ET, elephant trunk; FET, frozen elephant trunk; IPTW, inverse probability of treatment weighting.

*P* ≦ 0.05, statistical significance.

### After IPTW adjustment

No significant differences were observed between the two groups in demographic characteristics or preoperative malperfusion status. Cardiogenic shock occurred in approximately 18%–19% of the patients in the two groups. Compared with the AR group, the TAR group had significantly higher rates of primary arch entry site (70.3% vs 6.3%, *P* < 0.001), tear resection (86.3% vs 64.6%, *P* ≤ 0.001), and thoracic stent graft use (66.4% vs 24.1%, *P* ≤ 0.001). The TAR group also had longer cardiopulmonary bypass times (316.6 vs 262.0 min, *P* ≤ 0001), aortic cross-clamp times (168.1 vs 129.6 min, *P* ≤ 0001), and durations of circulatory arrest with ACP (56.3 vs 40.7 min, *P* ≤ 0001). Regarding postoperative outcomes, the TAR group had a higher incidence of AKI (32.3% vs 20.6%, *P* = 0.029), new-onset stroke (9.5% vs 3.7%, *P* = 0.016), and bleeding (30.1% vs 16.2%, *P* = 0.007) and had longer hospital stays (34.4 vs 25.7 days, *P* = 0.042) than did the AR group (Table 2).

### Long-term follow-up

**Figure 2.**
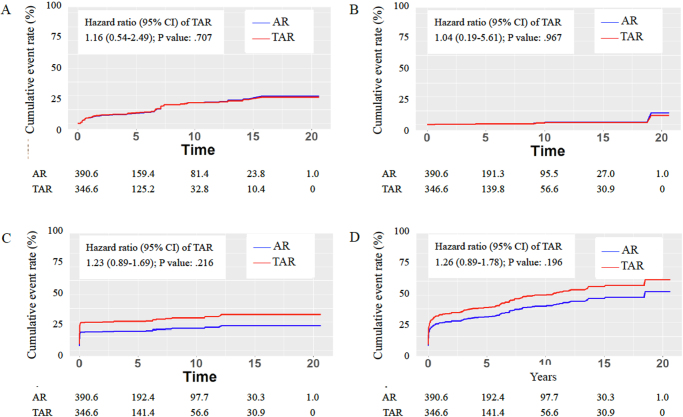
The cumulative incidence risk of patients undergoing total arch replacement (TAR) versus ascending aorta replacement (AR) in the IPTW-adjusted cohort. (A) Distal reintervention rate. (B) Proximal reintervention rate. (C) Aorta-related mortality rate. (D) Overall mortality rate. Blue line: AR; red line: TAR.

During long-term observation, no significant differences were noted in the rates of distal or proximal anastomotic reintervention events between the two groups (Fig. 2A and 2B). The subdistribution HR did not reveal a significant difference between TAR and AR (Table 3). Moreover, no significant differences were observed in the incidence of aorta-related mortality events between the two groups (Fig. 2C and Table 3). Regarding overall mortality events, no significant differences were noted between the AR and TAR groups (Fig. 2D). The HR analysis similarly did not reveal a significant difference (Table 3). In the time-stratified analysis, no significant differences were noted in subgroup comparisons. However, the overall mortality rate was higher in patients undergoing TAR after 2011 (Supplemental Digital Content Table S2, available at http://links.lww.com/JS9/F306; HR = 1.79, *P* = 0.017, *E* value = 2.98).

**Table 3:** Long-term outcome of patients undergoing aortic repair in the IPTW-adjusted cohort

	AR, *N* = 390.6	TAR, *N* = 346.6	HR or SHR (95% CI) of TAR	*P*-value	*P* for interaction
Distal reintervention	59.3 (15.2)	43.2 (12.5)	1.16 (0.54-2.49)	0.707	0.001
Proximal reintervention	5.5 (1.4)	3.0 (0.9)	1.04 (0.19-5.61)	0.967	–
Aorta-related mortality	73.4 (18.8)	96.8 (27.9)	1.23 (0.89-1.69)	0.216	0.655
Overall mortality	141.5 (36.2)	162.4 (46.9)	1.26 (0.89-1.78)	0.196	0.982

HR, hazard ratio; SHR, subdistribution hazard ratio; IPTW, inverse probability of treatment weighting.

*P* ≦ 0.05, statistical significance.

### Age-dependent HR for overall mortality

**Figure 3.**
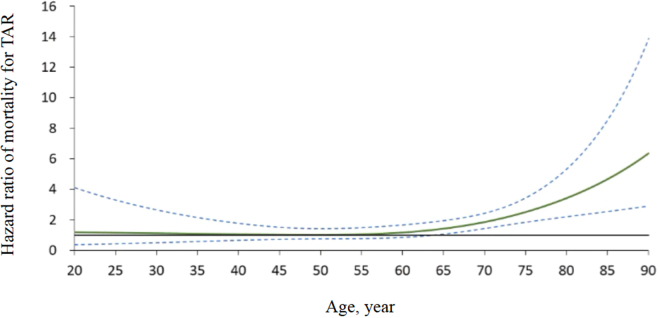
Age-dependent hazard ratio of overall mortality in patients undergoing total arch replacement (TAR) relative to ascending aorta replacement (AR) in the IPTW-adjusted cohort.

The analysis of overall mortality revealed a significant age-dependent effect after 65 years, with the effect based on the surgical method (Fig. 3). The risk of relative mortality associated with TAR was significantly higher in older patients.

## Discussion

Although higher rates of AKI, new-onset stroke, and bleeding and longer hospital stays were observed in patients who underwent TAR compared with those in patients who underwent AR, no significant differences were noted in 30-day mortality between the two groups. In the long-term observation, the rates of distal reintervention, proximal reintervention, aorta-related mortality, and overall mortality were similar between the groups. However, TAR was associated with a higher mortality risk than AR was in patients older than 65 years. These findings indicate that a tear-oriented policy for selecting between TAR and AR is appropriate in choosing lifesaving treatment in patients up to 65 years of age. However, the increased risk associated with TAR should be carefully considered in patients older than 65 years.

The baseline characteristics before IPTW were similar between the AR and TAR groups, with the exception of sex and preoperative cerebral malperfusion, the differences for which were of borderline significance (*P* = 0.046 and *P* = 0.040, respectively). After IPTW adjustment, these variables were well balanced (*P* = 0.639 and *P* = 0.767, respectively), indicating improved comparability between the surgical approaches.

The overall 30-day mortality rate was 18.2%, and the rate was 16.9% in the AR group and 20.3% in the TAR group. This result is comparable to the 18% reported in the NORCAAD database from 2005 to 2015 and the 16.9% reported in the GERAADA database from 2006 to 2010^[20,21]^. More recent data from the STS database indicated a 30-day mortality rate of 17% between 2014 and 2017[22]. These findings indicate that the present study’s results are acceptable and consistent with real-world outcomes. Regarding new-onset stroke, the overall incidence in this study was 6.8%, with incidences of 4.5% in the AR group and 10.5% in the TAR group. This overall incidence was lower than those reported in other databases. For example, an incidence of 15.7% was reported in the NORCAAD database, and one of 13.0% was reported in the STS database^[20,22]^. This may be because definitions of postoperative stroke rates have varied across studies. Cerebrovascular accident history and preoperative cerebral malperfusion were excluded to enable clearer comparison of new-onset stroke outcomes between the surgical groups. Regardless of the aforementioned differences, the stroke rates observed in this study are acceptable for long-term mortality comparisons. Notably, the higher stroke rate in the TAR group compared with the AR group can be explained by the longer clamping time and supra-aortic vessel manipulation during the TAR procedure, which may dislodge tiny particles of debris or aortic plaques into the brain[22].

In our study, a tear-oriented policy was applied for the treatment of acute DeBakey type I aortic dissection to reduce the incidence of late distal reoperation events. After IPTW adjustment, the tear resection rate remained higher in the TAR group than in the AR group. This study expected that the distal reintervention rate would be lower in the TAR group. However, the distal reintervention rate did not significantly differ between the TAR and AR groups during the long-term follow-up. This paradox may be explained by differing mechanisms underlying similar distal reintervention rates. In the AR group, distal reintervention was often required because of aneurysmal degeneration of the residual dissected aorta, a condition typically associated with persistent patent false lumen, regardless of proximal residual tear size, false lumen diameter, or initial descending aorta diameter compared with TAR. By contrast, reintervention in the TAR group was frequently caused by stent graft–related complications, such as stent graft-induced new entry (SINE) or deformation, which can create new tears and repressurize the false lumen. Notably, many TAR patients underwent hybrid procedures involving proximal relocation of the distal anastomosis, supra-aortic debranching, and elephant trunk repair with stent grafting into the descending aorta. Although this approach leads to better true lumen expansion and hemostasis, it also increases reliance on stent grafts, increasing the risk of stent-related complications. Some reinterventions were performed in the groups to address persistent distal false lumen, either by extending previous stent grafts or sealing re-entry sites, and were classified as events even when prophylactic. Although anatomical differences in the patent false lumen existed between the groups, the distinct failure mechanisms – aneurysmal progression in AR and device-related problems in TAR – may have offset each other, leading to comparable overall distal reintervention rates. This outcome may also explain the equivalent aorta-related mortality in our study when the distal aortic events were addressed in a timely manner, with it resulting from residual aneurysm in AR or stent graft–related complication in TAR. Lerdpunnapongse *et al* reported a similar reintervention rate to ours, but they did not specify the indications for distal reintervention, rendering comparison difficult[23]. In Omura *et al*, the rates of freedom from distal aortic surgery for aortic dilation did not significantly differ between AR and TAR. However, when aortic dilatation greater than 50 mm was considered, the difference became significant[24]. In the present study, the threshold for residual dissecting aneurysm requiring intervention was also more than 50 mm, suggesting that residual dissecting aneurysms remained more prevalent in AR. In addition, distal anastomotic leakage in AR may have led to persistent patent false lumen in arch, particularly in cases involving longer anastomoses during hemiarch aortic repair. This mechanism differs from the distal SINE induced by oversizing at the distal edge of the stent graft in TAR.

Our results are consistent with Mikko’s conclusion that primary tear resection alone does not determine midterm survival outcomes[14]. In our risk analysis for reintervention events, mortality was treated as a competing risk and was excluded to allow for a clearer evaluation of reintervention outcomes. In addition, aorta-related mortality was analyzed. Among the patients with aorta-related mortality in this study, reintervention was refused even when progressive residual dissection reached the surgical indication. No significant differences were noted between the TAR and AR groups. In summary, the similar rates of reintervention and aorta-related mortality suggest comparable aortic remodeling between the TAR and AR groups. Notably, the initially higher rates of aorta-related and overall mortality may reflect increased 30-day mortality in the TAR group. However, mortality rates between groups exhibited parallel trends thereafter.

Although age is not a contraindication for the surgical treatment of acute aortic dissection, the selection between TAR and AR remains a topic of debate because age is a major risk factor for poor surgical outcomes[25]. In studies by Qin and Komatsu, limited aortic resection was recommended for older patients with acute aortic dissection, with older patients defined as individuals older than 65 or 70 years, depending on the institution^[26,27]^. Higher rates of AKI, new-onset stroke, and longer cardiopulmonary bypass times were observed among the patients aged ≥65 years, although these differences were not associated with significantly higher 30-day mortality (Supplemental Digital Content Table S1, available at http://links.lww.com/JS9/F306). Notably, postoperative organ injury in this age group is often unpredictable. Orhan *et al* reported that prolonged bypass time may cause immunosuppression and sepsis, and Chen *et al* demonstrated that AKI can impair the pharmacokinetics of anesthetics and antibiotics in older adults^[28,29]^. Stroke, which can result from prolonged bypass time, may lead to irreversible sequelae. Poor stroke recovery in older patients compromises cough reflexes and walking ability, both of which are independent risk factors for late mortality in older adults with acute aortic dissection[30]. These factors may explain the higher overall mortality observed in patients aged ≥65 years during follow-up (Supplemental Digital Content Figure, available at http://links.lww.com/JS9/F305). Accordingly, we recommend that decision-making for aortic repair in older patients should prioritize stabilizing vital signs and minimizing complication risks to optimize survival outcomes instead of focusing on uncertain future aortic remodeling.

The cost-effectiveness of surgical approaches has rarely been discussed in the literature. In our study, the TAR group experienced higher rates of AKI, stroke, and bleeding and longer bypass time, which led to extended hospital days and, thus, higher admission costs. Furthermore, patients in the TAR group were more likely to require readmission because of postoperative sequelae, even if they were discharged after the initial admission. The frequent use of thoracic stent grafts in TAR also increased surgical costs compared with those required for AR. During long-term follow-up, although the overall rate of distal reintervention did not differ significantly between groups, the indications for reintervention in the TAR group were more often related to stent graft complications. This trend is consistent with findings from the AHA Economic Burden and Healthcare Resource database[31].

This study has several limitations, including its retrospective nature and small sample size. In proximal reintervention analysis, a wide confidence interval was observed in the subdistribution HR, indicating possible data sparsity or model instability. This finding should be interpreted with caution. In addition, the surgical techniques evolved during the study period, transitioning from traditional grafting to stent grafting, and postoperative care practices improved over time. These advancements, along with potential confounders and biases, may have affected outcomes. Therefore, additional studies with larger sample sizes are warranted.

## Conclusion

According to the present study for the management of acute DeBakey type I aortic dissection, no significant differences were observed between TAR and AR in terms of 30-day mortality, reintervention events, aorta-related mortality, or overall mortality. However, older patients undergoing TAR had poorer overall survival outcomes than did those undergoing AR, particularly among patients older than 65 years.

## Data Availability

Data supporting the findings of this study are available upon request from the corresponding author. These data are not publicly available because of privacy and ethical restrictions.

## Research registration unique identifying number (UIN)

Retrospective study, IRB number: 2022-02-011CC.
